# *De novo* venom gland transcriptomics of *Calliophis bivirgata flaviceps*: uncovering the complexity of toxins from the Malayan blue coral snake

**DOI:** 10.1590/1678-9199-JVATITD-2021-0024

**Published:** 2021-09-24

**Authors:** Praneetha Palasuberniam, Kae Yi Tan, Choo Hock Tan

**Affiliations:** 1 Venom Research and Toxicology Laboratory, Department of Pharmacology, Faculty of Medicine, University of Malaya. Kuala Lumpur, Malaysia.; 2 Department of Biomedical Sciences, University Malaysia Sabah, Faculty of Medicine and Health Sciences, Kota Kinabalu, Sabah, Malaysia.; 3 Protein and Interactomics Laboratory, Department of Molecular Medicine, Faculty of Medicine, University of Malaya. Kuala Lumpur, Malaysia.

**Keywords:** Three-finger toxins, Delta-elapitoxin-CB1a, Calliotoxin, Maticotoxin, Snakebite, Envenomation

## Abstract

**Background:**

The Malayan blue coral snake, *Calliophis bivirgata flaviceps*, is a medically important venomous snake in Southeast Asia. However, the complexity and diversity of its venom genes remain little explored.

**Methods:**

To address this, we applied high-throughput next-generation sequencing to profile the venom gland cDNA libraries of *C. bivirgata flaviceps*. The transcriptome was *de novo* assembled, followed by gene annotation, multiple sequence alignment and analyses of the transcripts.

**Results:**

A total of 74 non-redundant toxin-encoding genes from 16 protein families were identified, with 31 full-length toxin transcripts. Three-finger toxins (3FTx), primarily delta-neurotoxins and cardiotoxin-like/cytotoxin-like proteins, were the most diverse and abundantly expressed. The major 3FTx (Cb_FTX01 and Cb_FTX02) are highly similar to calliotoxin, a delta-neurotoxin previously reported in the venom of *C. bivirgata*. This study also revealed a conserved tyrosine residue at position 4 of the cardiotoxin-like/cytotoxin-like protein genes in the species. These variants, proposed as Y-type CTX-like proteins, are similar to the H-type CTX from cobras. The substitution is conservative though, preserving a less toxic form of elapid CTX-like protein, as indicated by the lack of venom cytotoxicity in previous laboratory and clinical findings. The ecological role of these toxins, however, remains unclear. The study also uncovered unique transcripts that belong to phospholipase A_2_ of Groups IA and IB, and snake venom metalloproteinases of PIII subclass, which show sequence variations from those of Asiatic elapids.

**Conclusion:**

The venom gland transcriptome of *C. bivirgata flaviceps* from Malaysia was *de novo* assembled and annotated. The diversity and expression profile of toxin genes provide insights into the biological and medical importance of the species.

## Background

Snake venoms consist of toxins that are primarily proteins and peptides with diverse pharmacological activities [1]. These toxins are products of venom evolution, representing successful traits critical for the survival of various snake species [2]. The evolvability of venom enables the snakes to adapt to different niches and, in turn, facilitates ecological speciation [3]. Worldwide, approximately 300 venomous snake species are considered medically important as they are implicated in snakebite envenomation, a life-threatening condition caused by the venom inoculated in snakebite victims [4, 5]. It is estimated that 1.8-2.7 million people are affected by snakebite envenomation, resulting in 81,000 to 138,000 deaths annually [5, 6].

In Asia, neurotoxicity is a typical manifestation of envenomation caused by elapid snakes such as cobras, king cobra, kraits and sea snakes. In addition, there is a unique clade of elapids, namely the Asiatic coral snakes, that are often considered less medically important due to their infrequent encounter with human and low fatality rate of envenomation [5]. As such, the venom properties of Asiatic coral snakes are generally less studied in comparison to most other elapids. This limits our understanding of the biological significance and potential application of venoms from these evolutionarily distinct coral snakes in Asia. 

**Figure 1.**
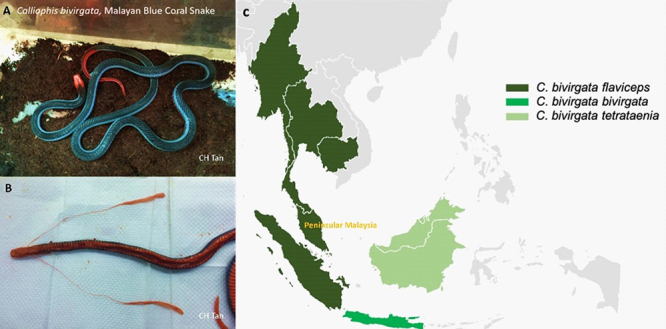
Morphology and distribution of Malayan blue coral snake (*Calliophis bivirgata*). **(A)** Dorsal view showing red coloration of the head and tail (which is continuous on the ventral surface), with the back in black or dark blue, flanked by a pair of alluring blue streaks alongside the body. **(B)** Dissection revealing a pair of exceptionally elongated venom glands in the snake. **(C)** Areas shaded in green depicting the native distribution of *Calliophis* subspecies in Southeast Asia based on the Reptile Database [16]. Photos by Choo Hock Tan.

The Asiatic (Old World) coral snakes are diverse, comprising three genera, *i.e.*, *Calliophis*, *Hemibungarus* and *Sinomicrurus* [7]. Among these, the blue coral snake (*Calliophis bivirgata*) is perhaps the most well-known and attractive owing to its unique morphology with striking coloration - its head, tail and underside (ventral surface) are red, and its back is dark blue to black in color flanked by a pair of alluring blue streaks alongside its body [8]. *C. bivirgata* has a pair of exceptionally long venom glands that extends beyond the jaw distally for one-third the length of the body (Figure 1), and it is ophiophagic (feeding on snakes) [9]. They are often fossorial, hiding beneath rainforest grounds and this makes them very elusive. Although *C. bivirgata* is often described as reclusive and less aggressive, it can be hostile and ready to bite when provoked. With its extraordinarily long venom glands, an adult *C. bivirgata* can yield a large amount of venom (~150 mg) in a single milking [10], implying potential medical complications upon severe envenomation [11-13].

*C. bivirgata* is endemic to Southeast Asia and three subspecies are recognized across different localities: *C. bivirgata flaviceps* in Thailand, Peninsular Malaysia, and Sumatra; *C. bivirgata tetrataenia* in Borneo; and *C. bivirgata bivirgata* in Java [14-16] (Figure 1 depicts the native range of the species in Southeast Asia). Among these subspecies, *C. bivirgata flaviceps* (found in Thailand and Peninsular Malaysia) has been reported to cause human fatalities [11, 12]. A recent proteomic study showed that Malaysian *C. bivirgata flaviceps* venom contains high amounts of cytotoxin-like proteins (22.6%) and phospholipases A_2_ (41.1%) which could be instrumental in the pathophysiology of envenomation [15]. Other studies further detected a sodium channel antagonist, a delta-neurotoxin called calliotoxin in *C. bivirgata* venom, indicating that *C. bivirgata* envenomation can potentially result in neurotoxicity [10, 17]. The *C. bivirgata* venom, however, showed negligible immunological cross-reactivity with various elapid antivenoms that were raised against non-coral snake species in Asia, despite having abundant three-finger toxins and phospholipases A_2_ as the venoms of most other elapid species [18]. These reports unveiled the real hidden threat in *C. bivirgata* envenomation: there is no effective antivenom available to treat the envenomation, and an inappropriate antivenom given to a patient may result in fatal hypersensitive reactions. 

The proteome and antigenicity of *C. bivirgata* venom indicate that the toxin genes of this species are evolutionarily divergent from the other elapids. This has implications on the medical importance of this species in terms of pathophysiology and treatment of envenomation. To better understand the diversity and functions of its toxins, this study investigated the *de novo* venom gland transcriptome of *C. bivirgata flaviceps* (a subspecies from Peninsular Malaysia) through a high-throughput next-generation sequencing approach and deep data mining. In addition, the findings were compared to a recent study of three-finger toxin evolution in this species which also employed the venom gland transcriptomic method [17]. Uncovering the complexity and the diversity of toxin genes in this unique species enriches our current knowledge base of snake venoms, and provides deeper insights into the clinical, biomedical, and evolutionary significance of the Asiatic coral snakes [19]. 

## Methods

### Preparation of snake venom gland tissue

The Malayan blue coral snake, *C. bivirgata flaviceps* was a male adult specimen from Pahang in the central region of Peninsular Malaysia. The snake was milked four days before venom gland removal to promote maximal transcription [20]. The venom glands were collected following euthanasia and sectioned into dimensions of 5 x 5 mm. The sectioned tissue was immersed in RNAlater Solution (Ambion, TX, USA) at 4 (C overnight followed by −80 (C until further use. The study was carried out in line with protocols approved by the Institutional Animal Use and Care Committee (IACUC) of University of Malaya, Malaysia (Approval code: #2013-11-12/PHAR/R/TCH).

### Extraction and purification of total RNA

The venom gland tissue was homogenized in a 1 ml glass homogenizer with TRIzol solution (Invitrogen, CA, USA), followed by adding 20% chloroform to separate the RNA (aqueous state) from the DNA and proteins (interface and organic states) [21]. The sample was centrifuged and treated with RNA-free DNAase I (Thermo Fisher Scientific, MA, USA), and the RNA was precipitated with isopropyl alcohol, followed by washing with 70% ethanol [22]. The quality of the purified total RNA was assessed using Agilent 2100 Bioanalyzer (Agilent RNA 6000 NanoKit) (Agilent Technologies, Waldbronn, Germany). The RNA integrity number of the sample was 8.9 (Grade A), indicating that the quality of RNA was in good condition for further downstream transcriptomic analysis. 

### Construction of cDNA library

The cDNA library construction was carried out with MGIEasy RNA Library Prep Set (Item No: 1000006383), as per manufacturer’s instructions. In brief, mRNA was purified with Dynabeads^®^ mRNA Purification Kit using magnetic beads. Enriched poly(A)^+^ mRNA isolated from the total venom gland RNA was adopted for cDNA construction. The isolated mRNA was fragmented into short fragments, which acted as templates for cDNA synthesis. Quantitative validation was conducted using ABI StepOnePlus Real-time PCR system (Applied Biosystem, CA, USA), which quantified adaptor-ligated sequences whereas qualitative validation was done using Agilent 2100 Bioanalyzer (Agilent Technologies, Waldbronn, Germany) for quality control of cDNA. The sample was then sequenced in a single lane on the BGISeq-500 platform (BGI Genomics Co., Ltd., Shenzhen, China) with 100-base-pair, paired-end reads.

### Filtration of raw sequenced reads

Sequenced data generated from BGISeq-500 platform were transformed into raw reads in a FASTQ format. Raw reads were filtered as part of the quality control process in the pre-analysis stage [23]. Raw reads with unknown bases of more than 5%, reads containing adaptor sequences and low-quality reads which possessed reads containing more than 20% bases with quality score less than 15 were removed before downstream transcriptome assembly. 

### Assembly of *de novo* transcriptome

The *de novo* ‘shot-gun’ transcriptome assembly was performed using a short-reads assembly program, Trinity version 2.0.6, which includes three software modules, Inchworm, Chrysalis, and Butterfly [24, 25], to reconstruct long scaffolds from single reads. Trinity was the assembly method of choice in this study as this program has been shown to recover good transcripts after passing quality filters while also recovering relatively long transcripts at its highest specificity [26, 27]. The clean read Q20 score, a point of reference for quality control assessment was obtained as a benchmark for successful *de novo* assembly of the transcriptome. 

### Clustering of transcripts

Unigenes obtained from Trinity were processed for sequence splicing and removal of redundant reads utilizing TIGR Gene Indices Clustering Tool (TGICL), version 2.0.6, to isolate non-redundant (NR) transcripts at the longest length [28]. Transcripts that shared nucleotide sequence similarity of more than 70% were grouped into clusters (transcript ID with the prefix CL labelled as contigs) whereby those sharing less than 70% similarity were labelled as singletons (transcript ID with a prefix of Unigene).

For functional annotation, all transcripts were subjected to BLASTx (Basic Local Alignment Search Tool-x) applying NCBI non-redundant database (NR), with a cut-off value of E <10^-5^. The coding regions of transcripts were determined by referencing to the highest-ranked proteins. 

### Quantifying transcript abundance

Clean reads were aligned to Unigene using Bowtie2, version 2.2.5 [29]. The gene expression levels were then calculated using RNA-seq with expectation maximization (RSEM) tool, version 1.2.12 [30]. Fragments per kilobase of exon model per million reads mapped (FPKM) were used to determine the transcript abundance for identified genes [31]. FPKM is the summation of normalized read counts based on gene length and the total number of mapped reads. The data was obtained using RSEM tool in conjunction with Trinity based on a computational formula:

$$FPKM\;of\;gene\;A\;=\frac{10^{6}B}{NC∕1000}$$

FPKM is the expression of gene A; B is the number of fragments/reads which are aligned to gene A; N is the total number of fragments/reads that are aligned to all genes; C is the base number in the coding sequence of gene A.

### Categorization of transcripts

The de novo assembled transcripts were subjected to BLASTx search to obtain the closest resembling sequences from the NR protein database for classification based on functional annotations. The transcripts (Unigenes) were then sifted to remove those with an FPKM value of less than 1, followed by categorization into three groups: “toxins”, “non-toxins” and “unidentified” as previously described [32-34] (Additional file 1). “Toxin” transcripts were recruited by toxin-related keywords search against the annotated transcripts [33, 35, 36]. “Non-toxin” and “unidentified” groups contain transcripts of cellular proteins or house-keeping genes, and transcripts that could not be identified, respectively. The redundancy of gene expression was determined by dividing the total FPKM of each group by the total number of transcripts in the respective group of transcripts [34]. In the toxin group, the amino acid sequences were used to further validate the toxin identity through BLASTp suite (Basic Local Alignment Search Tool-Protein) in the UniProtKB (Universal Protein Resource Knowledgebase) database platform. The transcripts were searched against Serpentes database (taxid: 8570) and validated based on the lowest E-score value with the highest percentage of sequence similarity (updated as of March 23, 2020). The transcripts and sequences, clustered into different toxin families, and information of full-length transcripts, were provided in Additional file 2.

### Multiple sequence alignment

Multiple sequence alignment was conducted using Jalview software (version 2.11.1.4) [37] and MUSCLE (Multiple Sequence Comparison by Log-Expectation) [38] program on amino acid sequences of toxins. Sequences of related species used in multiple sequence alignment were retrieved from UniProtKB depository (http://www.uniprot.org/). The sequences and species selected for comparison were based on toxinological relevance and purpose to elucidate similarity and variation between comparing sequences. 

### Selection pressure analysis

Sequences of interest were aligned using Mega X (version 10.0.5). Selection pressure was then analyzed using pairwise distances with amino acid substitution and Maximum Composite Likelihood model. Non-synonymous and synonymous (dN/dS) substitution rates were obtained. 

## Results and discussion

### Sequencing and de novo transcriptome assembly

De novo sequencing of the cDNA library of the Malayan blue coral snake (C. bivirgata flaviceps) venom gland tissue yielded a total of 50,850,478 clean reads. Assembly of the de novo short reads created 79,142 contigs (N50 = 1,640) that were connected to form 52,809 transcripts (N50 = 1,906). Output and quality metrics of RNA sequencing were provided in Additional file 3. The accuracy of sequencing indicative of successful de novo venom gland transcriptome was validated by the base call accuracy of Q20 percentage at 98.53%. 

**Figure 2.**
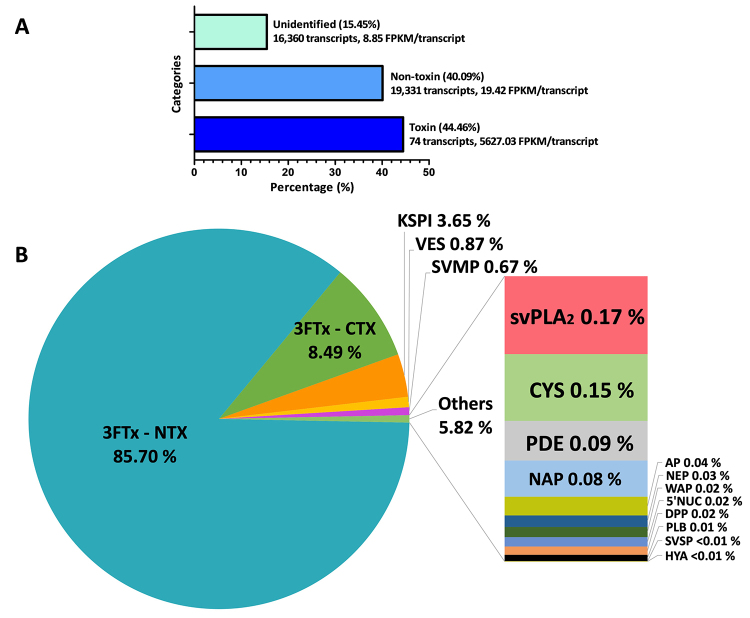
Venom gland transcriptome of *Calliophis bivirgata flaviceps* from Malaysia. **(A)** Abundance (in percentage of total FPKM) and redundancy (average FPKM/transcript) of transcripts according to “toxin”, “non-toxin” and “unidentified” categories. **(B)** Expression profile of toxin transcripts according to protein families (in percentage of total toxin FPKM). The percentages indicate the relative abundances of transcripts based on expression levels in FPKM, as outlined in the method. FPKM: fragments per kilobase of transcript per million mapped reads; 3FTx: three-finger toxin; NTX: neurotoxin; CTX: cytotoxin; KSPI: Kunitz-type serine protease inhibitor; VES: vespryn; SVMP: snake venom metalloproteinase; svPLA_2_: snake venom phospholipase A_2_; CYS: cystatin; PDE: phosphodiesterase; NAP: natriuretic peptide; AP: aminopeptidase; NEP: neprilysin; WAP: waprin; DPP: dipeptidyl peptidase; 5’NUC: 5’nucleotidase; PLB: phospholipase B; SVSP: snake venom serine protease; HYA: hyaluronidase.

The transcripts were filtered at the FPKM (fragments per kilobase per million mapped reads) of ≥ 1 and yielded 35,766 transcripts. Based on BLASTx search, the transcripts were assigned into three categories: (a) “toxin”; (b) “non-toxin”; and (c) “unidentified” (Table 1; Figure 2A). Transcripts in the “toxin” category encoded known and putative snake toxins, and these dominated the transcriptome by a total FPKM of 44.46%. Transcripts in the “non-toxin” category encoded proteins with no known toxic activities in envenomation, and these constituted 40.09% of the total FPKM. Meanwhile, the “unidentified” category (15.45% of total FPKM) included transcripts with no identifiable hits from the BLASTx search. The high expression of toxin transcripts (dominating virtually half of the venom gland transcriptome) in the venom gland of this species is consistent with observations reported in Micrurus spp. (American coral snakes) whose toxin gene transcription accounted for 46-61% of the venom gland transcriptome [39, 40].

**Table 1:** Overview of transcript classification and expression in de novo venom gland transcriptome of *C. bivirgata flaviceps*.

Total number of transcripts at fragments per kilobase of exon model per million mapped reads (FPKM) > 1	35,766		
Categories	**Toxin**	**Non-toxin**	**Unidentified**
Number of transcripts	74	19,331	16,360
Relative abundance of transcripts (% of total FPKM)	44.46	40.09	15.45
Redundancy (average FPKM/transcript)	5,627.03	19.42	8.85

### Expression of toxin genes in C. bivirgata flaviceps venom gland

A total of 74 distinct transcripts were identified in the “toxin” category. These transcripts were clustered by sequence similarities and relevance of snake venom function into 16 different families of toxin genes (Table 2). Although the total number of all toxin transcripts was only 74, the high expression of all toxins contributed to an exceptionally high redundancy (on average, 5,627.03 FPKM/toxin transcript) in contrast to the much lower levels noted in the non-toxin and unidentified gene groups (19.42 FPKM/transcript and 8.85 FPKM/transcript, respectively) (Figure 2A), consistent with the high activity of toxin gene transcription in the venom gland tissue of the snake.

Out of the 74 toxin transcripts, 31 transcripts have ≥ 90% sequence coverage based on the annotated protein entries. These toxins were classified under full-length transcripts in the present study (Table 2; sequences available in Additional file 2). The majority of toxin transcripts were annotated based on sequence similarities from various elapid species, while only four transcripts were matched to sequences specific to *Calliophis*species, indicating that the existing toxin database is indeed limited for the Asiatic coral snakes. 

Among the 16 toxin gene families, three-finger toxins (3FTx) are the most diversified and abundantly expressed (20 transcripts at 94.19% of total toxin FPKM). Its domination in the venom gland transcriptome suggests that the 3FTxs are critical in the venom evolution of *C. bivirgata flaviceps* and implicated in its adaptation to specific ophiophagic diet. The diversely expressed 3FTx genes in this species were further categorized by sequence subtypes into long-chain, short-chain and non-conventional groups (Table 2), and elaborated based on their functional attributes in the context of envenomation. Other toxin families were expressed at much lower levels. These include Kunitz-type serine protease inhibitor (KSPI), vespryn (VES), snake venom metalloproteinase (SVMP), snake venom phospholipase A_2_ (svPLA_2_), cystatin (CYS), phosphodiesterase (PDE), natriuretic peptide (NAP), aminopeptidase (AP), neprilysin (NEP), waprin (WAP), 5’ nucleotidase (5’NUC), dipeptidyl peptidase (DPP), and phospholipase B (PLB), snake venom serine protease (SVSP) and hyaluronidase (HYA). Figure 2B illustrates the overall venom gland transcriptome of *C. bivirgata flaviceps* according to the toxin gene families.

**Table 2.** Overview of toxin gene families and subtypes in the venom gland transcriptome of *Calliophis bivirgata flaviceps*.

Toxin gene families/subtypes	Species and annotation based on sequence similarities		Transcript abundance^a^ (non-redundant transcript)
**Three-finger toxin (3FTx)**			**94.19 (20)**
**Short-chain 3FTx**			**94.16 (18)**
Delta-elapitoxin-Cb1a^*^	P0DL82	*Calliophis bivirgatus*	40.14 (2)
Three-finger toxin MALT0070C	F5CPE6	*Micrurus altirostris*	20.07 (3)
Neurotoxin 3FTx-LI	P0C553	*Bungarus fasciatus*	13.68 (3)
Cytotoxin A5^*^	P62375	*Naja atra*	7.26 (2)
Neurotoxin 3FTx-RK	P0C554	*Bungarus fasciatus*	5.87 (1)
Three-finger toxin D.L	A0A0H4BLZ2	*Micrurus diastema*	4.63 (2)
Three-finger toxin T.B	A0A0H4IS80	*Micrurus tener*	1.21 (1)
Maticotoxin A^*^	P24742	*Calliophis bivirgatus*	1.15 (1)
Cytotoxin homolog 3^*^	P01473	*Naja melanoleuca*	0.07 (1)
Neurotoxin 3FTx-RI	P0C555	*Bungarus fasciatus*	0.04 (1)
Neurotoxin-like protein NTL2	Q9W717	*Naja atra*	0.03 (1)
**Long-chain 3FTx**			**0.03 (1)**
Long neurotoxin LlLong	Q7T2I3	*Laticauda laticaudata*	0.03 (1)
**Non-conventional 3FTx**			**< 0.01 (1)**
Probable weak neurotoxin 3FTx-Lio1	A7X3M9	*Erythrolamprus poecilogyrus*	< 0.01 (1)
**Phospholipase A_2_ (svPLA_2_)**			**0.17 (5)**
Phospholipase A_2_ 2^*^	P24645	*Calliophis bivirgatus*	0.08 (1)
Basic phospholipase A_2_ PC17^*^	Q8UUH8	*Laticauda colubrina*	0.07 (2)
Basic phospholipase A_2_ PC9	Q8UUH9	*Laticauda colubrina*	0.02 (1)
Group XV phospholipase A_2_	V8NS07	*Ophiophagus hannah*	< 0.01 (1)
**Snake venom metalloproteinase (SVMP)**			**0.67 (10)**
Asrin^*^	A6XJS7	*Austrelaps superbus*	0.50 (1)
Snake venom metalloproteinase-disintegrin-like mocarhagin	Q10749	*Naja mossambica*	0.10 (1)
Zinc metalloproteinase-disintegrin-like MTP4	F8RKW1	*Drysdalia coronoides*	0.04 (2)
Zinc metalloproteinase-disintegrin-like ohanin	A3R0T9	*Ophiophagus hannah*	0.01 (1)
Nigrescease-1	B5KFV8	*Cryptophis nigrescens*	0.01 (2)
Zinc metalloproteinase-disintegrin-like NaMP^*^	A8QL59	*Naja atra*	< 0.01 (1)
Zinc metalloproteinase-disintegrin-like BmMP	A8QL49	*Bungarus multicinctus*	< 0.01 (1)
Zinc metalloproteinase-disintegrin-like atragin	D3TTC2	*Naja atra*	< 0.01 (1)
**Kunitz-type serine protease inhibitor (KSPI)**			**3.65 (4)**
Kunitz-type serine protease inhibitor mulgin-3	Q6ITB9	*Pseudechis australis*	3.16 (1)
Kunitz-type serine protease inhibitor textilinin-4	Q90W98	**Pseudonaja textilis textilis**	0.47 (1)
Kunitz-type serine protease inhibitor vestiginin-2	A6MFL2	*Demansia vestigiata*	0.02 (1)
Kunitz-type protease inhibitor 1^*^	V8N7R6	*Ophiophagus hannah*	0.01 (1)
**Vespryn (VES)**			**0.87 (1)**
Ohanin^*^	P83234	*Ophiophagus hannah*	0.87 (1)
**Cystatin (CYS)**			**0.15 (6)**
Cystatin^*^	E3P6N7	*Pseudechis porphyriacus*	0.12 (1)
Cystatin-C^*^	V8NX38	*Ophiophagus hannah*	0.01 (1)
Cystatin-B	V8P5H9	*Ophiophagus hannah*	0.01 (1)
Cystatin 1	A0A2H4N3F5	*Bothrops moojeni*	< 0.01 (2)
Cystatin^*^	E3P6P4	*Naja kaouthia*	< 0.01 (1)
**Phosphodiesterase (PDE)**			**0.09 (2)**
Snake venom phosphodiesterase	5GZ4	*Naja atra*	0.05 (1)
Venom phosphodiesterase 1	J3SEZ3	*Crotalus adamanteus*	0.04 (1)
**Natriuretic peptides (NAP)**			**0.08 (2)**
Natriuretic peptide Na-NP	D9IX97	*Naja atra*	0.08 (1)
C-type natriuretic peptide	Q09GK2	*Philodryas olfersii*	< 0.01(1)
**Aminopeptidase (AP)**			**0.04 (9)**
Aminopeptidase^*^	B6EWW5	*Gloydius brevicaudus*	0.03 (2)
Aminopeptidase NPEPL1^*^	A0A2H4N3C8	*Bothrops moojeni*	0.01 (2)
Aminopeptidase^*^	T2HQN1	*Ovophis okinavensis*	< 0.01 (2)
Aminopeptidase B^*^	V8N861	*Ophiophagus hannah*	< 0.01 (1)
Aminopeptidase O	V8NPW5	*Ophiophagus hannah*	< 0.01 (2)
**Neprilysin (NEP)**			**0.03 (1)**
Neprilysin^*^	V8NQ76	*Ophiophagus hannah*	0.03 (1)
**Waprin (WAP)**			**0.02 (2)**
Porwaprin-b^*^	B5L5N2	*Pseudechis porphyriacus*	0.02 (1)
Waprin-Phi3^*^	A7X4M7	*Philodryas olfersii*	< 0.01 (1)
**5' nucleotidase (5’NUC)**			**0.02 (3)**
Snake venom 5' nucleotidase^*^	F8S0Z7	*Crotalus adamanteus*	0.02 (1)
5' nucleotidase^*^	A6MFL8	*Demansia vestigiata*	< 0.01 (1)
5' nucleotidase	A0A024AXW5	*Micropechis ikaheca*	< 0.01 (1)
**Dipeptidyl peptidase (DPP)**			**0.02 (5)**
Dipeptidyl peptidase 1^*^	V8N9E5	*Ophiophagus hannah*	0.01 (1)
Dipeptidyl peptidase 2^*^	V8NF35	*Ophiophagus hannah*	0.01 (1)
Dipeptidyl peptidase 9^*^	V8NC40	*Ophiophagus hannah*	< 0.01 (2)
Dipeptidyl peptidase 8^*^	V8N5Q6	*Ophiophagus hannah*	< 0.01 (1)
**Phospholipase B (PLB)**			**0.01 (2)**
Phospholipase-B 81^*^	F8J2D3	*Spilotes sulphureus*	0.01 (1)
Phospholipase B-like	V8NLQ9	*Ophiophagus hannah*	< 0.01 (1)
**Snake venom serine protease (SVSP)**			**< 0.01 (1)**
Snake venom serine protease	P18965	*Daboia siamensis*	< 0.01 (1)
**Hyaluronidase (HYA)**			**< 0.01 (1)**
Hyaluronan and proteoglycan link protein 3	V8P471	*Ophiophagus hannah*	< 0.01 (1)

a Transcript abundance expression (%) is based on FPKM (fragments per kilobase of exon model per million reads mapped). ^*^Number of full-length toxin transcripts from venom gland of *Calliophis bivirgata flaviceps*. Determined by the coverage of amino acids of transcripts to amino acids of mature chain of annotated proteins.

### Three-finger toxins (3FTxs)

3FTxs are typically the major toxins of elapids, e.g., cobras [41, 42], kraits [43, 44], sea snakes [45], mambas [46, 47] and coral snakes [39, 48, 49]. These are non-enzymatic polypeptides with molecular weights of approximately 6-9 kDa, orientated in three beta-stranded loops that resemble three protruding fingers [50, 51]. Comparison of the venom gland transcriptomic profiles between the current study and a recent report [17] reveals a similar dominating pattern of 3FTx, notwithstanding variation in the relative proportions of many toxin subtypes (Table 3). The variation implies potential inter-individual differences between the specimens, e.g., wild versus captive snakes from different geographical regions, or extensive post-translational modifications [52]. Variations also could be accounted for by methodological differences, in terms of tissue harvesting time [20] and program algorithms used in the study. Although transcriptional events in venom gland was shown to achieve the highest level four days after venom extraction [20], the transcriptome may represent only a snapshot of toxin gene expression as different genes likely have varying transcription rates and mRNA half-lives. 

**Table 3.** Comparison of venom gland transcriptomics of the Malayan blue coral snake (*Calliophis bivirgata*) between two studies.

Toxin gene families	Current study^a^	Dashevsky et al. [17]^b^
Total transcripts	74	125
Three-finger toxins	**20,** 94.16%	**67,** 82%
Neurotoxins	**16,** 85.70%	NS
Cytotoxin-like proteins	**4,** 8.49%	NS
Kunitz-type serine protease inhibitor	**4,** 3.65%	**24,** 9%
Phospholipase A_2_	**5,** 0.17%	**13,** 8%
Vespryn	**1,** 0.87%	**1,** ^*^
Snake venom metalloproteinase	**10,** 0.67%	**4,** ^*^
Cystatin	**6,** 0.15%	**4,** ^*^
Phosphodiesterase	**2,** < 0.1%	**1,** ^*^
Natriuretic peptide	**2,** < 0.1%	NR
Aminopeptidase	**9,** < 0.1%	NR
Neprilysin	**1,** < 0.1%	**1,** ^*^
Waprin	**2,** < 0.1%	**1,** ^*^
5’Nucleotidase	**3,** < 0.1%	NR
Dipeptidyl peptidase	**5,** < 0.1%	NR
Phospholipase B	**2,** < 0.1%	**1,** ^*^
Snake venom serine protease	**1,** < 0.01%	NR
Hyaluronidase	**1,** < 0.01%	**1,** ^*^
Cysteine-rich secretory proteins	NR	**2,** ^*^
Kallikrein	NR	**1,** ^*^
Nerve growth factors	NR	**4,** ^*^

Bold indicates the number of transcripts in each toxin gene family and protein subtype. Percentage indicates the relative abundance of transcripts. ^a^Relative abundance of transcripts based on fragments per kilobase per million mapped reads (FPKM). ^b^Relative abundance of transcripts based on transcripts per million (TPM). ^*^One of the 11 toxin gene families with their total abundance reported as < 1% collectively. NS: not specified; NR: not reported.

**Figure 3.**
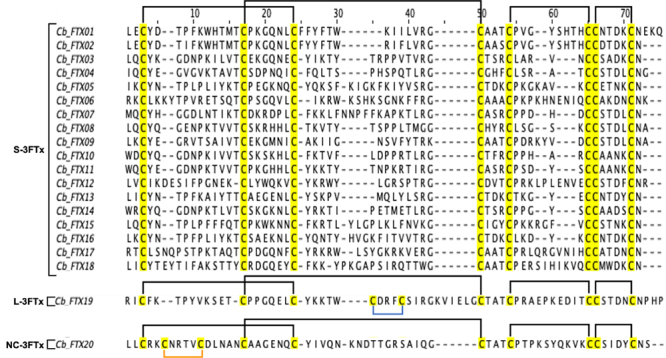
Multiple sequence alignments of three-finger toxin (3FTx) transcripts from *Calliophis bivirgata flaviceps*. Black brackets denote the four disulfide bridges in all 3FTx, while blue and orange brackets represent the fifth disulfide bridges present in L-3FTx (second loop) and NC-3FTx (first loop), respectively.

Based on the protein structure, 3FTx transcripts in *C. bivirgata flaviceps* venom gland transcriptome were further categorized into short-chain 3FTx (S-3FTx, with four disulfide bridges), long-chain 3FTx (L-3FTx, with an additional fifth disulfide bridge on the second loop) and non-conventional 3FTx (NC-3FTx, with an additional fifth disulfide bridge on the first loop) [51]. Within the 3FTxs family, the most abundantly and diversely expressed transcripts belonged to S-3FTxs (94.16%, 18 transcripts), while L-3FTx and NC-3FTx each consisted of only one lowly expressed transcript (< 0.05% of total toxin FPKM) (Table 2). Of these, the majority of S-3FTx transcripts (40.14% of toxin FPKM, two transcripts) were annotated by sequence similarities to a delta-neurotoxin, called calliotoxin (UniProtKB: P0DL82). Other S-3FTx composed of 12 putative neurotoxins or neurotoxin-like proteins and four cardiotoxin-like/cytotoxin-like proteins. Multiple sequence alignment revealed highly conserved four disulfide bridges (formed by eight conserved cysteine residues) among the 18 S-3FTx, and five disulfide bridges in each of the L-3FTx and NC-3FTx respectively (Figure 3). 

### Short-chain three-finger toxins (S-3FTxs)

*Delta-neurotoxins (calliotoxin)*

**Figure 4.**
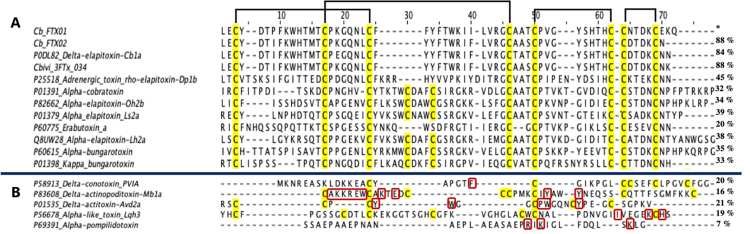
Multiple sequence alignments of Cb_FTX01 and Cb_FTX02 of *Calliophis bivirgata flaviceps* compared to functionally matched sequences from **(A)** serpentes and **(B)** non-serpentes groups obtained from public database. Percentage indicates the sequence similarities compared to Cb_FTX01(^*^). Black brackets: disulfide bridges, red regions: critical binding amino acid residues.

In the venom gland transcriptome, Cb_FTX01 and Cb_FTX02 were the two most abundantly expressed transcripts of S-3FTx. Both transcripts were annotated as calliotoxins (UniProtKB: P0DL82 and Cbivi_3FTx_034 reported by Dashevsky et al. [17]), which is by far the only delta-neurotoxin discovered from snake venoms [10]. To elucidate variation in the genes, sequences of Cb_FTX01 and Cb_FTX02 were further compared with calliotoxins and representative alpha-neurotoxins from common elapid species found in Southeast Asia (Figure 4). Both Cb_FTX01 and Cb_FTX02 exhibited high sequence similarities (88-100%) to calliotoxins (UniProtKB: P0DL82) and Cbivi_3FTx_034 [17], suggesting similar pharmacological properties of these delta-neurotoxins expressed in the venom gland transcriptome. An earlier study showed that calliotoxin binds to the sodium channels of motor neurons, prolonging the action potential and resulting in spastic paralysis [10]. The delta-neurotoxin is presumably the principal toxin that causes neurotoxic death in C. bivirgata envenomation, and this neurotoxic effect has been shown to affect different taxa including mammals and birds [10, 15] but resist neutralization by elapid antivenoms [18]. 

On the other hand, the delta-neurotoxins of *C. bivirgata flaviceps* lack the specific binding sites toward the alpha-subunit of nicotinic acetylcholine receptor (nAChR) (Lys-27, Trp-29 and Asp-31) [53], and have very low sequence similarity (20-45%) to elapid alpha-neurotoxins (Figure 4). The emergence of delta-neurotoxin in *C. bivirgata flaviceps* venom marks the divergent evolution of its 3FTx from virtually all other elapids, whose neurotoxic 3FTxs are primarily alpha-neurotoxins. Alpha-neurotoxins block the nAChR and cause flaccid paralysis as the mode of kill, as opposed to spastic paralysis induced by delta-neurotoxins. In nature, delta-neurotoxins are typically present in the venoms of invertebrate animals, e.g., scorpions, solitary wasps, cone snails, spiders, and sea anemone [54-58]. In snakes, thus far, delta-neurotoxins were discovered only from this species. The delta-neurotoxins of *C. bivirgata flaviceps*, nonetheless, are distinct with only 7-21% of sequence similarities to those of the invertebrate animals. Comparing across different taxa, the delta-neurotoxins of *C. bivirgata flaviceps* are highly divergent from the following invertebrates at their respective critical binding sites: Leiurus hebraeus (UniProtKB: P56678, at Ile-59, Lys-64, His-66) [59]; Anoplius samariensis (UniProtKB: P69391, at Arg1, Lys3 or Lys12) [60]; Conus purpurascens (UniProtKB: P58913, at Phe-60 and Ile-63) [61]; Missulena bradleyi (UniProtKB: P83608, at Lys-3, Lys-4, Arg-5, Glu-6, Trp-7, Lys-10, Glu-12, Tyr-22, Tyr-25) [62]; Anemonia sulcata (UniProtKB: P01535, at Tyr-7, Trp-8, Pro-12, Trp-13 and Tyr-18) [63] (Figure 4). This illustrates a case of functional convergence where *C. bivirgata flaviceps* and these unrelated species, in their respective biomes, have independently evolved toxins that are functionally similar for the purpose of prey paralyzing. 

*Cardiotoxin-like/Cytotoxin-like proteins*

**Figure 5.**
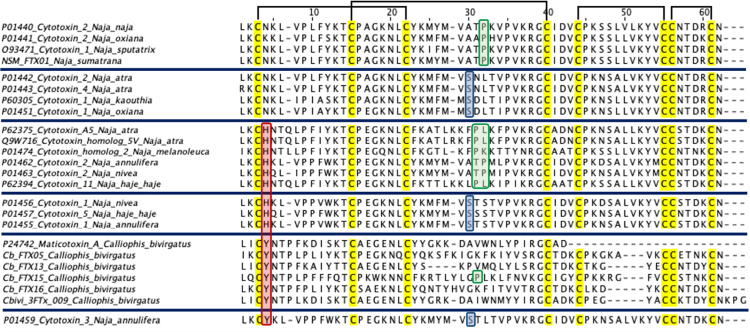
Multiple sequence alignments of cytotoxin-like transcripts of *Calliophis bivirgata flaviceps* (Cb_FTX05, -13, -15, -16) aligned and compared to closely related sequences from public database. Highlighted in: red box - His-4/Tyr-4-type; blue box - Ser-28-type, green box - Pro-31-type; black brackets - disulfide bridges.

Within the S-3FTx group, Cb_FTX05, Cb_FTX13, Cb_FTX15 and Cb_FTX16 are four transcripts encoding for cytotoxin-like/cardiotoxin-like proteins, and accounting for 8.49% of the total toxin FPKM (Table 3; Additional file 2). Cb_FTX05 and Cb_FTX15 were annotated as cytotoxin (CTX) homologues from *Naja atra* (UniProtKB: P62375, with 53-55% similarities), and Cb_FTX16 was annotated as one from Naja melanoleuca (UniProtKB: Q9W716, with 51% similarity) (Figure 5). Although the CTX-like proteins of *C. bivirgata flaviceps* were annotated as those from cobra (Naja spp.), they were found to have only ~50% sequence similarity, indicating that the CTX-like proteins of *C. bivirgata flaviceps* are evolutionarily divergent from the cobra CTX, and thus these are purely putative toxins whose activities were hypothesized based on sequence similarities. Significant variations were observed particularly in the loop II region which is critical for CTX binding to the lipid bilayer of membrane [51] (Figure 5), suggesting that the CTX-like proteins of the coral snake are functionally varied from those of cobras.

The other CTX-like transcript, Cb_FTX13, was annotated as maticotoxin (UniProtKB: P24742), a cardiotoxin-like protein reported previously in C. bivirgata venom [15, 64]. Maticotoxin exhibited weak cytotoxicity in vitro but caused hemolysis in synergistic action with phospholipase A_2_ [15, 64]. Earlier, in the report by Takasaki et al. [64], maticotoxin was only partially sequenced to 41 amino acid residues with the use of Edman degradation method. The present study, together with the recent study by Dashevsky et al. [17], successfully uncovered its full amino acid sequence and the presence of eight conserved cysteine residues (forming four disulfide bridges) that are responsible for its three-fingered structure (Figure 5). Noteworthy, all CTX-like proteins from *C. bivirgata flaviceps* represent a unique class not conforming to the usual classification of CTX known presently. Chien et al. [65] through studying the binding effect of various cobra CTX to lipids, proposed that CTX could be divided into P-type and S-type based on the presence of Pro-31 or Ser-29 in the sequence. Both amino acid residues are located within the same phospholipid binding sites in the tip of loop 2, but Ser-29 is located in the more hydrophilic region (thus weaker binding to lipid membrane) [65]. A variant H-type, whose 4^th^ amino acid residue is substituted with histidine, was noted in some CTX regardless of P-type or S-type, and these “H-type” CTX variants are generally less toxic due to weak membrane binding activity [66]. The CTX-like sequences of *C. bivirgata flaviceps* contain no distinguishable conserved amino acid at residue positions 29 and 31 (neither Ser-29 nor Pro-31) but, interestingly, display conserved tyrosine at the 4th residue. The emergence of Tyr4 in these sequences could be probably a result of His substitution by Tyr involving a single nucleotide mutation from C to U at the codon (CAU/CAC to UAU/UAC). The mean dN/dS ratio comparing the H-type CTX cytotoxin A5 and homolog 5V of cobra to the CTX-like proteins of *C. bivirgata flaviceps* are 0.60 and 0.95, respectively, implying a synonymous substitution, and that the evolution of the Tyr-4-containing CTX variant is near neutral or under constraint. Considering that His and Tyr were both polar, aromatic and having a positive amino acid substitution similarity score (+2 based on BLOSUM 62 scoring matrix), the replacement is likely conservative, thus preserving a less cytotoxic form of maticotoxin in the *C. bivirgata flaviceps* venom [15, 64] as with the H-type CTX of cobra [66]. The exact ecological role of the Try-4-containing variants, which we propose as the Y-type CTX/CTX-like proteins of elapid snakes, remains to be further investigated.

### Other three-finger toxins (3FTxs)

A total of 12 transcripts, constituting 45% of the toxin FPKM, were annotated to various neurotoxin-related genes (Table 4). These transcripts were matched with limited sequence similarities mainly to S-3FTXs reported from coral snakes (Micrurus spp.), cobras (Naja spp.), kraits (Bungarus spp.) and sea krait (*Laticauda* spp.). Notably, most of these transcripts were only present at the mRNA level (present study) but not detected in the venom proteome reported earlier [15], suggesting a complex regulatory process between gene transcription and protein translation. Some of the transcripts probably undergo rapid degradation, pre-empting meaningful translation as in pseudogenization - a more common fate for a duplicated gene, which would have been anticipated in the course of evolution of the extended 3FTx gene family in this species. Are these representations of pseudogenes conserved among the elapids, evolving free of selective pressure and following random genetic drift? Regardless, these minor proteins if translated in the venom would be toxins that potentially play an integral role in the overall venom function. The biological function and pathophysiological roles of these toxins, nevertheless, deserve further investigation in the future. 

**Table 4.** Other putative neurotoxins in *C. bivirgata flaviceps* venom gland transcriptome.

Toxin transcripts	Toxin subtypes	Annotated sequences and species	Reference	Sequence similarities (%)	Toxin FPKM (%)
***Short-chain three-finger toxins***					
Cb_FTX03 Cb_FTX08 Cb_FTX09	Three-finger toxin MALT0070C	F5CPE6, *Micrurus altirostris*	[39]	42	20.1
				41	
				41	
Cb_FTX04 Cb_FTX07 Cb_FTX14	Neurotoxin 3FTx-LI	P0C553, *Bungarus fasciatus*	[67]	41	13.7
				43	
				42	
Cb_FTX06	Neurotoxin 3FTx-RK	P0C554, *Bungarus fasciatus*	[68]	65	5.9
Cb_FTX10 Cb_FTX11	Three-finger toxin D.L	A0A0H4BLZ2, *Micrurus diastema*	Uncharacterized	42	4.6
				45	
Cb_FTX12	Three-finger toxin T.B	AKO63249, *Micrurus tener*	Uncharacterized	55	1.2
Cb_FTX17	Neurotoxin_3FTx-RI	P0C555, *Bungarus fasciatus*	[67]	44	<0.1
Cb_FTX18	Neurotoxin-like protein NTL2	Q9W717, *Naja atra*	Uncharacterized	44	<0.1
***Long-chain three-finger toxins***					
Cb_FTX19	Long neurotoxin LlLong	Q7T2I3, *Laticauda laticaudata*	[69]	93	<0.1
***Non-conventional three-finger toxins***					
Cb_FTX20	Probable weak neurotoxin 3FTx-Lio1	A7X3M9, *Erythrolamprus poecilogyrus*	Uncharacterized	60	<0.1

### Snake venom phospholipases A_2_ (svPLA_2_)

**Figure 6.**
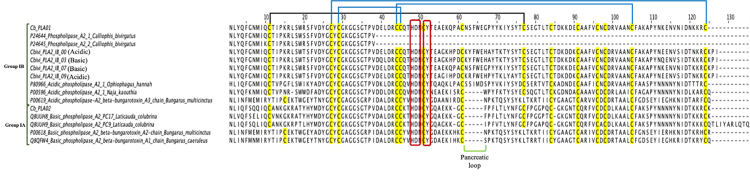
Multiple sequence alignment of snake venom phospholipase A_2_ (svPLA_2_) transcripts (Cb_PLA01 and Cb_PLA02) of *Calliophis bivirgata flaviceps* aligned and compared to closely related sequences from public database. Brackets in blue and green: conservative disulfide bonds and residues of pancreatic loop; black: additional disulfide bridges; red box: conserved amino acid residues.

Snake venom phospholipases A_2_ are extensively distributed in the venoms of virtually all snake species, although recent studies revealed exceptions in the venoms of African non-spitting cobras (subgenus: Uraeus) [70, 71]. Conventionally, snake venom PLA_2_ (svPLA_2_) are classified into the secretory svPLA_2_ family of Groups IA (cobras and kraits), IIA (most viperids) and IIB (Gaboon vipers) [72]. In the *C. bivirgata flaviceps* venom gland transcriptome, a total of five svPLA_2_ transcripts (Cb_PLA01-05, 0.17 % of total toxin FPKM) were identified (Table 2; Additional file 2). Of these, the full-length transcripts of the two main svPLA_2_ (Cb_PLA01 and Cb_PLA02) were characterized, and categorized as Group IB and Group IA svPLA_2_, respectively. Conserved amino acid residues His-48, Tyr-52 and Asp-99 in svPLA_2_ [72] were present in the svPLA_2_ transcripts of *C. bivirgata flaviceps*. In addition to these critical amino acid residues, these svPLA_2_ transcripts contain Asp-49 which is one of the key residues for enzymatic activities [723]. Similarly, Cb_PLA01 and transcripts retrieved from another study (Cbivi_PLA2_00, -03, -07 and -09) [17] contain a surface loop located between residues 62 to 66, termed pancreatic loop, which is a characteristic of Group IB svPLA_2_ [74] (Figure 6). Group IB svPLA_2_, mainly found in mammalian pancreas, have been reported in only a few snake venoms from elapids such as Oxyuranus scutellatus [75], *Pseudonaja textilis* [76] and *Micrurus frontalis frontalis* [77]. The presence of the hydrophilic pancreatic loop in Group IB svPLA_2_ has been shown to decrease the substrate-PLA_2_ enzyme binding activity [74], but the biological significance of this phenomenon and its effect on snakebite envenomation remain unclear. In comparison, Cb_PLA01 exhibited high sequence similarities to Cbivi_PLA2_00, -03, -07 and -09 (86-99% sequence similarities), suggesting they may exhibit similar pharmacological activities. It is also notable that Group IA svPLA_2_ (Cb_PLA02) was absent in the recently reported study of the same species [17].

Based on the computation determination of theoretical pI (isoelectrical point) using ExPASy computational tool (SIB Swiss Institute of Bioinformatics), Cb_PLA01, Cbivi_PLA2_IB_00 and Cbivi_PLA2_IB_09 isoforms were found to be acidic (pI: 6.70, 5.88 and 5.88, respectively) whereas Cb_PLA02, Cbivi_PLA2_IB_03 and Cbivi_PLA2_IB07 isoforms were basic (pI: 8.75, 8.19, and 7.53, respectively). On multiple sequence alignment, Cb_PLA01 were annotated as acidic svPLA_2_ from *Ophiophagus hannah* (UniProtKB: P80966) and *Naja kaouthia* (UniProtKB: P00596) with sequence similarities of ~70% (Figure 6). An acidic svPLA_2_ from king cobra was previously reported to cause myocardial and skeletal muscle degeneration in mice [78], while acidic svPLA_2_ from other elapids generally lack toxicity [45, 79, 80]. On the other hand, Cb_PLA02 (coding for a basic svPLA_2_) showed ~90% sequence similarity to two basic svPLA_2_ isoforms from the sea krait, *Laticauda colubrina* (UniProtKB: Q8UUH8 and Q8UUH9) (Figure 6). Multiple sequence alignment in Figure 6 further revealed that both svPLA_2_ transcripts (CB_PLA01 and CB_PLA02) have limited sequence similarity (45−50%) to the pre-synaptic beta-bungarotoxins from Bungarus spp. (kraits), which are also ophiophagic venomous snakes found in Asia. The finding is consistent with transient myotoxicity and the lack of presynaptic neurotoxic activity caused by *C. bivirgata flaviceps* venom [15, 64].

### Snake venom metalloproteinase (SVMP)

Snake venom metalloproteinases (SVMP) are multi-domain proteins with diverse biological activities, e.g., causing hemorrhage, fibrinogenolysis and defibrination, and inhibition of platelet aggregation [81]. These proteins are usually major components in the venoms of vipers and pit vipers, consistent with the pathological phenotypes seen in viper/pit viper envenomation (hemorrhagic syndrome and consumptive coagulopathy) [82]. Most elapid venoms contain little or small amount of SVMP, with the exceptions of king cobra [32] and Asian coral snakes (*Calliophis*spp.) [15, 17, 49]. The biological and pathological role of SVMP in elapid venoms has not been well elucidated although it could be contributing to inflammatory responses [83]. Interestingly, elapid SVMP are commonly members of PIII class which are more structurally and functionally variable [84]. In the case of *C. bivirgata flaviceps* venom gland transcriptome, a total of eight transcripts of SVMP, with two of which showing full-length sequences (Cb_SVMP01 and Cb_SVMP07), were identified at a very low abundance (0.7% of total toxin FPKM) (Table 2; Additional file 2). 

**Figure 7.**
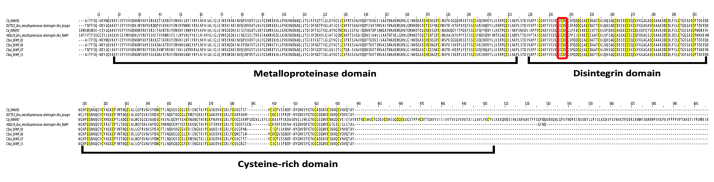
Multiple sequence alignment of snake venom metalloproteinase (SVMP) transcripts (Cb_SVMP01 and Cb_SVMP07) of *Calliophis bivirgata flaviceps* with other annotated sequences. Brackets in black: metalloproteinase, disintegrin and cysteine-rich domains; red regions: ECD motif.

All the transcripts from the present work and a recent study [17] belong to PIII class of SVMP in which the mature chain of protein consists of the metalloproteinase, disintegrin-like, and cysteine-rich domains, with conserved ECD (glutamic acid, cysteine, aspartic acid) integrin-binding motifs present at the disintegrin domain (Figure 7). In comparison, Cb_SVMP01 showed high sequence similarities (82-94%) to transcripts Cbivi_SVMP02, -06, -07 and -11 (Figure 7) [17]. On BLAST search, the SVMP sequences of *C. bivirgata flaviceps* matched most closely to those from cobras (Naja spp.) but with limited sequence similarity (56−75%) (Figure 7), suggesting molecular and perhaps functional adaptation in SVMP that is unique to the Asiatic coral snake and divergent from the cobras. 

### Other minor toxin transcripts

Other toxin genes with low expression (< 5% of toxin FPKM) include Kunitz-type serine protease inhibitor (KSPI), vespryn (VES), cystatin (CYS), phosphodiesterase (PDE), natriuretic peptide (NAP), aminopeptidase (AP), neprilysin (NEP), waprin (WAP), 5’nucleotidase (NUC), dipeptidyl peptidase (DPP), phospholipase B (PLB), snake venom serine protease (SVSP) and hyaluronidase (HYA). Among these, VES, CYS, PDE, NUC and HYA were previously reported in the venom proteome at low abundances [15]. In contrast, the other toxin families, i.e., KSPI, NAP, AP, NEP, WAP, DPP, PLB and SVSP were not found in the venom proteome, presumably due to their very low protein abundances below the detection limit of the mass spectrometry used. From the venom gland transcriptomics, the findings suggest that the toxin genes are conserved in the *Calliophis*lineage. 

Meanwhile, a discrepancy was observed between the transcript expression level (present study) and protein abundance in the venom proteome reported previously [15]. The lack of correlation between toxin gene expression and venom protein abundance has also been reported in several earlier studies [32, 33, 39, 40, 85]. It should be noted that in most venom gland transcriptomic studies, the venom gland transcriptome reflects a “snapshot” of gene expression at a certain time point when the gland tissue was harvested, typically a few days after venom milking. The mRNAs of various toxins could be expressed at different rates, having varying half-lives and subjected to complex regulation processes including post-transcriptional and post-translational modifications [86]. These events in between could have further modulated the maturation and secretion of proteins into the final venom product, thus the lack of correlation between the transcript expression and protein abundance. Furthermore, the use of a single de novo assembly program (Trinity) is a limitation in this study, as ideally a combination of multiple assemblers may have added advantage to fully capture the entirety of the transcripts expressed [26]. Nevertheless, Trinity (assembler program used in this study) is one that has been widely applied in venom-gland transcriptomics, and it has been shown to recover the highest number of good transcripts that passed quality filters [26, 27]. 

Furthermore, while calliotoxin (a delta-neurotoxin) has been identified in the present transcriptomic study and from the venom of the homologous Malaysian species [10], this unique toxin was not detected in the venom proteomics reported earlier [15]. Previously, the toxin was not detected in the venom proteome presumably due to the lack of the complete sequence back then to support a comprehensive reference search database. The proteomic approach adopted was deriving quantitative information from peptide-centric mass spectrometry data, which has its weakness in protein identification without a model organism to provide a reference database with complete sequence coverage. In regard to this, we propose that the venom proteome can be re-investigated using a more comprehensive database, incorporating the diverse species-specific toxin sequences including the calliotoxin isolated by Yang et al. [10], and de novo sequences from the recent study [17] and current work.

## Conclusion

To summarize, this study reported the de novo venom gland transcriptomics of *Calliophis bivirgata flaviceps*, a unique Asiatic coral snake species from the Peninsula of Malaysia. The findings unveiled the complexity and diversity of venom genes in the species, demonstrating inter-specific and intra-specific variations in the sequences and expressions of toxin genes. Of note, three-finger toxins uncovered from this species show remarkable variability in their sequences and putative functions from those of many Asiatic elapids, as indicated by the presence of delta-neurotoxins (including calliotoxin), Tyr-4-containing CTX-like proteins, svPLA_2_ of Group IA and IB, and divergent forms of PIII class of SVMP. The findings enriched the toxin knowledge base for the Malayan blue coral snake, and the comprehensive transcriptomic profiling provides deeper insights into the medical and biological significance of the species.

### Abbreviations

cDNA: complementary deoxyribonucleic acid; 3FTx: three-finger toxin; CTX: cytotoxin; C. bivirgata: *Calliophis bivirgata*; *C. bivirgata flaviceps*: *Calliophis bivirgata flaviceps*; FPKM: fragments per kilobase per million mapped reads; BLAST: basic local alignment search tool; KSPI: Kunitz-type serine protease inhibitor; VES: vespryn; SVMP: snake venom metalloproteinase; svPLA_2_: snake venom phospholipase A_2_; CYS: cystatin; PDE: phosphodiesterase; NAP: natriuretic peptide; AP: aminopeptidase; NEP: neprilysin; WAP: waprin; 5’NUC: 5’ nucleotidase; DPP: dipeptidyl peptidase; PLB: phospholipase B; SVSP: snake venom serine protease; HYA: hyaluronidase; NTX: neurotoxin; TPM: transcript per million; ND: not detected; NS: not specified; S-3FTx: short three-finger toxin; L-3FTx: long three-finger toxin; NC-3FTx: non-conventional three-finger toxin; nAChR: nicotinic acetylcholine receptor; mRNA: messenger ribonucleic acid; spp.: species pluralis; IACUC: Institutional Animal Use and Care Committee; RNA: ribonucleic acid; PCR: polymerase chain reaction; NCBI: National Centre for Biotechnology Information; NR: non-redundant; RSEM: RNA-seq with expectation maximization; BLASTp: Basic Local Alignment Search Tool-Protein; UniProtKB: Universal Protein Resource Knowledgebase; MUSCLE: Multiple Sequence Comparison by Log-Expectation. 

## Supplementary material

Additional file 1. Venom gland transcriptomic analysis of *Calliophis bivirgata flaviceps*.

Additional file 2. Classification and sequences of toxin genes from *Calliophis bivirgata flaviceps* venom gland transcriptome.

Additional file 3. Output and quality metrics of RNA sequencing for the de novo assembly of *Calliophis bivirgata flaviceps* venom gland transcriptome.
