# Supplementary material for: De novo venom gland transcriptomics of Calliophis bivirgata flaviceps: uncovering the complexity of toxins from the Malayan blue coral snake
Source: J Venom Anim Toxins Incl Trop Dis. 2021 Sep 24;27:e20210024. doi: 10.1590/1678-9199-JVATITD-2021-0024 (PMC8476087; doi:10.1590/1678-9199-JVATITD-2021-0024)
Supplement: Additional file 3. [file 1678-9199-jvatitd-27-e20210024-s3.pdf]

Journal of Venomous Animals and Toxins including Tropical Diseases
https://doi.org/ 10.1590/1678-9199-JVATITD-2021-0024

Supplementary Material to "*De novo* venom gland transcriptomics of *Calliophis bivirgata flaviceps*: uncovering the complexity of toxins from the Malayan blue coral snake"

Additional file 3. Output and quality metrics of RNA sequencing for the *de novo* assembly of *Calliophis bivirgata flaviceps* venom gland transcriptome.

Total raw reads	60,588,311
Total clean reads	50,850,478
Contigs created	79,142
N50	1,640
Number of unigenes/contigs assembled	52,809
N50	1,906
Q20 percentage	98.53
N percentage	1.5
GC percentage	43.71
